# Distribution characteristics of gastric mucosal colonizing microorganisms in different glandular regions of Bactrian camels and their relationship with local mucosal immunity

**DOI:** 10.1371/journal.pone.0300316

**Published:** 2024-05-30

**Authors:** Jianfei Li, Fie Xie, Xueyan Wang, Wangdong Zhang, Cuicui Cheng, Xiuping Wu, Min Li, Xingmin Huo, Xin Gao, Wenhui Wang

**Affiliations:** College of Veterinary Medicine, Gansu Agricultural University, Lanzhou, P.R.China; Wrocław University of Environmental and Life Sciences: Uniwersytet Przyrodniczy we Wroclawiu, POLAND

## Abstract

Bactrian camels inhabiting desert and semi-desert regions of China are valuable animal models for studying adaptation to desert environments and heat stress. In this study, 16S rRNA technology was employed to investigate the distribution characteristics and differences of mucosal microorganisms in the anterior gland area, posterior gland area, third gland area, cardia gland area, gastric fundic gland area and pyloric gland area of 5-peak adult healthy Bactrian camels. We aimed to explore the possible reasons for the observed microbial distribution from the aspects of histological structure and mucosal immunity. *Bacteroides* and *Fibrobacteria* accounted for 59.54% and 3.22% in the gland area, respectively, and 52.37% and 1.49% in the wrinkled stomach gland area, respectively. The gland area showed higher abundance of *Bacteroides* and *Fibrobacteria* than the wrinkled stomach gland area. Additionally, the anterior gland area, posterior gland area, third gland area, and cardia gland area of Bactrian camels mainly secreted acidic mucus, while the gastric fundic gland area mainly secreted neutral mucus and the pyloric region mainly secreted a mixture of acidic and neutral mucus. The results of immunohistochemistry techniques demonstrated that the number of IgA+ cells in the anterior glandular area, posterior glandular area, third glandular area, and cardia gland area was significantly higher than that in the fundic and pyloric gland area (*p* < 0.05), and the difference in IgA+ between the fundic and pyloric gland area was not significant (*p* > 0.05). The study revealed a large number of bacteria that can digest and degrade cellulose on the mucosa of the gastric gland area of Bactrian camels. The distribution of IgA+ cells, the structure of the mucosal tissue in the glandular region, and the composition of the mucus secreted on its surface may have a crucial influence on microbial fixation and differential distribution.

## 1. Introduction

Researchers have discovered that host-microbe interactions are crucial for normal mammalian metabolism and immune homeostasis [1–7]. The host provides the basis for the nutrition and survival of gastrointestinal microorganisms, which in turn aid in the digestion and absorption of nutrients by the host [8]. Many scholars have utilized 16S rRNA technology to investigate the diversity of microorganisms in the gastrointestinal tract of animals, and they have found that a multitude of different species of microorganisms exist in the gastrointestinal tract. Once the composition and function of these intestinal microorganisms become imbalanced, it can result in gastrointestinal, neurological, respiratory, metabolic, hepatic, and cardiovascular diseases [9]. The symbiotic relationship between ruminants and gastrointestinal microbiota allows the host to extract energy from the rumen’s recalcitrant lignocellulosic food [10]. *Fibrobacteria* are known as the primary cellulose degraders of the rumen microbiota and play a critical role in the degradation of low-quality fibers [11, 12]. Despite the low abundance (< 1%) of camel rumen *Fibrobacteria*, over 10% of cellulase and 4.7% of hemicellulase originate from *Fibrobacteria*, indicating their significant role in the rumen [13, 14]. Studies have demonstrated that the colonization of certain bacterial species, such as *Prevotella_1*, *Treponema_2*, and *Rikenellaceae_RC9*, in the rumen may be linked to their cellulose-degrading ability and carbohydrate metabolism [15]. In 1980, some scholars proposed the three-group theory of rumen microorganisms, which categorizes rumen microorganisms into three groups: flora in the rumen fluid, flora attached to the feed pellets, and flora attached to the rumen wall [16]. It should be noted that research on the diversity of microorganisms in animal digestive tracts has focused on the diversity of bacteria in rumen fluid or rumen contents, with few studies reporting on the bacterial communities that are fixed on the gastric mucosa.

The mucosal immune system (MIS) in the intestine is mainly composed of intestine-associated lymphoid tissue. Based on their functions, the MIS can be divided into two parts: the intestinal mucosal epithelium and lamina propria with diffuse distribution and the "induction site" of immune response, which includes Peyer’s node in the small intestine, mesenteric lymph nodes, large intestine, and rectal solitary lymphoid follicles. Pan cells located at the base of the crypt in the small intestinal epithelium can secrete antimicrobial peptides in response to bacteria or pathogens in the intestine, contributing to intestinal defense [17]. The mucus layer and antimicrobial peptides (AMPs) form a mucosal barrier against invasion by commensal bacteria, which directly affects the gut microbiota [18–20]. Secretory IgA is mainly distributed in the oral cavity, intestinal digestive juice, respiratory mucus, and colostrum. IgA in the intestinal digestive fluid is mainly produced and secreted by IgA-positive plasma cells in the intestinal mucosal lamina propria and usually exists in the form of dimers, known as secretory IgA (SIgA). Functionally, IgA molecules prevent direct contact between harmful pathogenic microorganisms in the intestinal lumen and the organism’s intestinal epithelial cells, playing a crucial role in maintaining the mucosal immune barrier [21]. Specifically, SIgA binds to the surface of external pathogenic microorganisms, wraps them, and forms a negatively charged hydrophilic group that is carried by the mucosal surface cells, Mucin glycoproteins with the same charge are repelled, preventing them from adsorbing onto the surface of mucosal cells. This physically protects the body from the invasion of external pathogenic microorganisms [22]. Additionally, SIgA can also actively bind to the flagella of some bacteria, neutralizing the bacterial enzymes and toxins secreted by the bacteria, hindering bacterial movement and related biological functions, and preventing them from passing through the mucosal epidermis, providing physical protection from the invasion and infection of external pathogenic microorganisms [23]. It should be noted that IgA also has a regulatory role in protecting the organism from external pathogens, which includes altering bacterial motility, regulating gene expression of intestinal bacteria, and helping some intestinal bacteria to colonize [24]. While most studies have focused on the intestinal tract, the interrelationship between mucosal immunity and quorum microorganisms in the gastric glandular region of Bactrian camels has not been reported.

**Fig 1:**
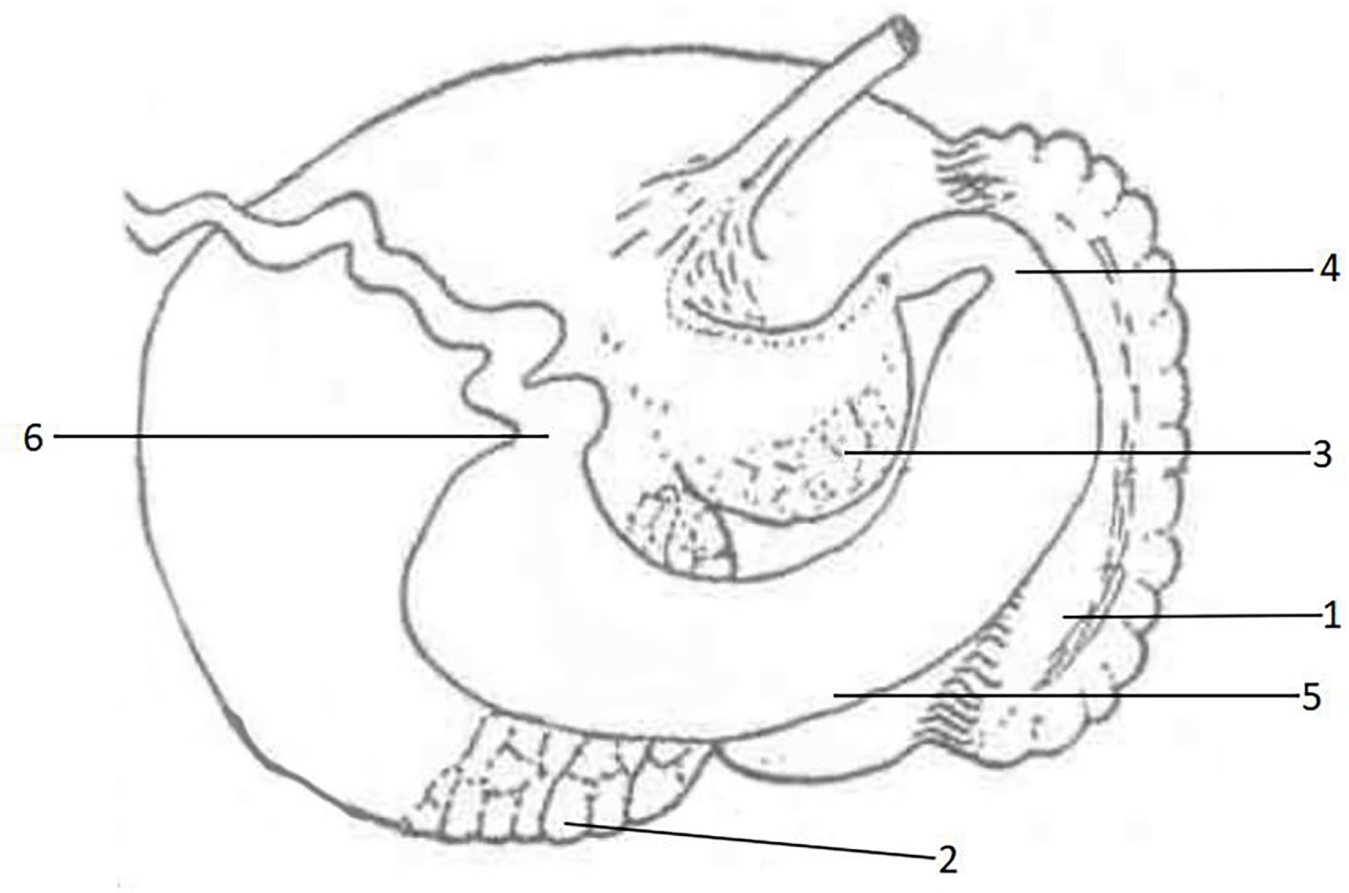
Gross anatomy of the bactrian camel stomach [26]. 1: Anterior gland area; 2: Posterior gland area; 3: Third gland area; 4: Cardia gland area; 5: Gastric fundic gland area; 6: Pyloric gland area.

The Alashan Bactrian camel primarily inhabits the desert and semi-desert regions of Inner Mongolia and Gansu in China, which are characterized by harsh living conditions. Through long-term natural selection, this species has evolved a unique stomach structure distinct from other ruminants [25]. Traditional ruminants, such as cattle, sheep, and deer, possess a four-part stomach composed of the rumen, reticulum, omasum, and abomasum, with the first three parts referred to as the antral stomach, lacking glandular distribution, and the abomasum possessing different glandular distribution. In contrast, the stomach of Bactrian camels is divided into the anterior stomach and the abomasum, with the anterior stomach divided into the first and second compartments by the interventricular sulcus, which are equivalent to the rumen and reticulum. The abomasum is divided into anterior enlargement, gastric body enlargement, and posterior enlargement. The anterior enlargement and gastric body are equivalent to the omasum, while the posterior enlargement is equivalent to the abomasum. The stomach of camelids features three compartments, with the first chamber being the largest and having two glandular regions known as the anterior and posterior glandular areas, and the second chamber being smaller and containing a glandular region located in the abdomen referred to as the third glandular area (Fig 1) [26]. The third chamber has a long tubular shape similar to that of the omasum in cattle and sheep [27]. The pancreatic gland area in Bactrian camels is around 70~80% of the total area of the abomasal mucosa, which is significantly larger than that in other animals, such as 33% in pigs, 7% in dogs, and 6% in rabbits [28]. In contrast, the fundic gland area in Bactrian camels accounts for only 11%~20% of the total area of the abomasal mucosa [29], which is smaller than that in other animals, such as 40% for pigs, 70% for dogs, and 64% for rabbits. The pyloric gland area accounts for 5%~6% of the total area of the abomasal mucosa, indicating that the unique gastric gland area of Bactrian camels may play an essential role in digesting cellulose. Research has shown that camel stomach digestion is more efficient than other ruminants, possibly due to its larger mucus-secreting glandular area [30]. Liu Shengwang, et al [31] have demonstrated that the glandular area is a unique anatomical structure of Bactrian camels that is not found in other ruminants, and its distinctive glandular area may aid in food digestion.

Although studies on bacterial diversity in the rumen contents and rumen fluid of camels and other ruminants have been conducted, studies on the diversity of mucosal colonizing microorganisms in the gastric glandular region of Bactrian camels have not been reported [32, 33]. The aim of this study is to investigate the distribution characteristics, distribution differences, and possible causes of bacterial communities on the mucosa of various glands in the stomach of Bactrian camels, laying a foundation for further exploration of the possible functions of bacterial communities in the food digestion of Bactrian camels.

## 2. Materials and methods

### 2.1 Ethics statement

All experimental procedures were approved by the Animal Care and Use Committee (IACUC) of College of Veterinary Medicine of Gansu Agricultural University (Approval No:GSAU-AEW-2016-0005).

### 2.2 Experimental design and sample collection

These camels are adult (3~5 years) healthy neutered camels from Minqin, Gansu, China. Sample collection immediately after slaughter in local abattoirs. The anterior glandular capsule region, posterior glandular capsule region, third glandular capsule region, cardia gland, pyloric gland and fundic gland were opened sequentially, and then the mucus was quickly scraped from the mucosal surface of each Bactrian camel under aseptic manipulation and quickly placed into 2.5 mL freezing tubes and stored in liquid nitrogen for further use. Then samples were collected and stored in 2.5% glutaraldehyde and 4% paraformaldehyde for electron microscopy and histological analysis solutions respectively and brought back to the laboratory for further use. A total of 30 samples were collected from the above six parts of five Bactrian camels.

### 2.3 Extraction of genome DNA

The total genome DNA was extracted from samples using the TIANamp Stool DNA Kit(TIANGEN, China) according to manufacturer’s instructions. 1% agarose gels were used to detect the integrity and impurity in the extracted bacterial DNA samples. The purity and concentration were tested by NanoPhotometer spectrophotometer (IMPLEN, Munich, Germany) and Qubit 2.0 Flurometer (Life Technologies, Carlsbad, CA, USA), respectively. According to the concentration, DNA was diluted to 1 ng/μL using sterile water.

### 2.4 PCR amplification and sequencing

The V3-V4 hypervariable regions of the bacteria 16S rRNA gene were amplified with primers 27F (5’-AGAGTTTGATCMTGGCTCAG-3’) and 1,492 (5’-TACGGYTACCTTGTTACGACTT-3’) on the GeneAmp1 PCR System 9,700 (Applied Biosystem, USA). All PCR reactions were carried out in 30 μL reactions with 15 μL of Phusion1 High-Fidelity PCR Master Mix (Biolabs, New England). 0.2 μm of forward and reverse primers, and about 10 ng of template DNA. Thermal cycling was consisted of initial denaturation at 95˚C for 3 min, followed by 25 cycles of denaturation at 95˚C for 30 s, annealing at 55˚C for 30 s, and elongation at 72˚C for 30 s, finally 72˚C for 5 min. The same volume of 1× loading buffer (contained SYB green) was mixed with PCR products and electrophoresis on 2% agarose gel was operated for detection. Samples with bright main strip about 460 bp (V3 ~ V4) were chosen for further experiments. PCR products were mixed in equidensity ratios. Then the mixture of PCR products were purified with GeneJET Gel Extraction Kit (Thermo Scientific, Waltham, MA, USA). Sequencing libraries were generated using NEB Next1 UltraTM DNA Library Prep Kit for Illumina (NEB,USA) following manufacturer’s recommendations and index codes were added. The library quality was assessed on the Qubit@ 2.0 Fluorometer (Life Technologies, CA, USA) and Agilent 2,100 Bioanalyzer (Agilent Technologies, Palo Alto, CA). At last, the library was sequenced on an Illumina MiSeq platform and 250 bp paired-end reads were generated. All the sequencing data were deposited in the National Center for Biotechnology Information Sequence Read Archive (NCBI SRA) under accession no. PRJNA888937.

### 2.5 16S rRNA high-throughput sequencing and data processing

Based on the Illumina MiSeq PE300 (Illumina, USA) sequencing platform, 16S rRNA high-throughput sequencing was performed on the bacterial genomes of different glandular regions of Bactrian camel on the basis of the same sample concentration and sequencing. The raw reads from Miseq sequencing were first stitched according to the overlap relationship, and the quality of the sequence was quality-controlled and filtered. After sequence filtering, sequences with high similarity (≥ 97%) were classified as an Operational Taxonomic Unit (OTU). Species classification analysis was performed in the RDP (Ribosomal Database Project) database using the blast comparison program. Based on the above taxonomic information, Rarefaction curves, Ace, Chao flora abundance index, and Shannon, Simpson bacterial diversity index were estimated. The data between the groups was statistically analyzed using SPSS20.0 software. The significance of differences between groups was tested using the Tukey post hoc method in one-way analysis of variance (ANOVA). A significance level of *p* < 0.05 was considered statistically significant.

### 2.6 Transmission electron microscopy fabrication

Appropriately sized tissue blocks were taken from different glandular regions of Bactrian camels using scissors and forceps, dehydrated, embedded, trimmed, sectioned, spread, and observed electron microscopically: After reaching a suitable field of view, increase the magnification and take individual images. Imaging is performed in InlensDUO mode with an EHT of 2 kv, a probe current of 5~10 pA and an aperture size between 30~60 μm. When capturing the image, keep the scanning speed 6~9, the resolution is 307232304 pixels, and use the line integral method to reduce noise.

### 2.7 Production of paraffin sections

The fixed gastric glandular tissues were sampled separately in the laboratory (size about 1 × 1 × 0.5 cm), put into the histological cassette and washed with PBS, then dehydrated by gradient alcohol in order (50%, 80%, 95%Ⅰ, 95% Ⅱ, 100% Ⅰ, 100% Ⅱ time in order of 2 h, 2 h, 2 h, 2 h, 1 h, 1 h), clarified by xylene, paraffin wax embedding and inclusion. After, subsequent experiments were performed.

### 2.8 Hematoxylin-eosin staining (H&E), Alcian blue-periodic acid sthiff (AB&PAS) staining

Cut and dried sections (thickness 4 μm), sequentially dehydrated by xylene, benzene alcohol and alcohol gradient, hematoxylin staining for 6 min, acid-water differentiation, eosin staining for 5 min after returning to blue, gradient alcohol dehydration, xylene transparency, neutral gum sealing, subsequent capture images; AB&PAS staining: conventional dewaxing, rinsing in tap water for 5 min, transferring to distilled water for 2 immersions, 3 min; Alcian blue staining for 40 min, soaking in distilled water for 2 times, 3 min; in oxidizing agent, placed at room temperature for 10 min; rinsing in tap water for 5 min, transferring to distilled water for 2 immersions, 3 min; placed in Schiff Reagent, placed in a dark place at room temperature. Immersion (plus 2 times) 20 min; tap water rinse 10 min; hematoxylin staining solution 2 min; tap water rinse 5 min; acid water fractionation 2~5 s; tap water rinse 10 min; ethanol conventional dehydration, transparent, seal the film.

### 2.9 Immunohistochemical staining

Sections were routinely gradient dewaxed; transferred to distilled water and washed for 5 min; thermal repair liquid (Tris-EDTA) started timing for 15 min when boiling and washed for 5 min × 3 times in distilled water after natural cooling. Incubate dropwise with Biotin Blocking Buffer for 60 min; wash with PBS for 5 min x 3 times,; incubate dropwise with Streptavidin/Biotin-Binding Blocking Buffer for 60 min; wash with PBS for 5 min × 3 times; with antibodies (diluted 1:100) against CD79a (GTX04701, GeneTex, USA) and incubate overnight (about 18 h); wash with PBS for 5 min × 3 times; add 3% methanol hydrogen peroxide for 40 min at room temperature; wash with PBS washing for 5 min × 3 times;

Immunohistochemistry plus horseradish peroxidase-labeled secondary antibody, incubated at 37°C for 40 min; washed with PBS for 5 min×3 times; DAB chromogenic solution for light microscopy, hematoxylin light staining for 1 min, acid-water differentiation for 3 s, tap water rinsing for 10 min, conventional dehydration, transparent and sealing.

### 2.10 Data analysis

Statistical analyses were performed using SPSS 22.0 (IBM Corporation, Armonk, NY, USA). Quantitative data are presented as the mean ± standard error of the mean. All data were tested for normality and homoscedasticity and subjected to one-way analysis of variance followed by Duncan’s multiple range test. Statistical significance was set at *p* < 0.05.

## 3. Results

### 3.1 OTU PCA and NMDS analysis

Thirty Bactrian samples of Bactrian camel had 1,075,578 Tags after removal of primers. The average number of samples was 35,825 with an average length of 419 bp. A total of 1,724 OTUs were generated from 30 samples. Species complexity analysis was performed based on the results of OTU and species annotation, and between-group species differences were analyzed.

**Fig 2:**
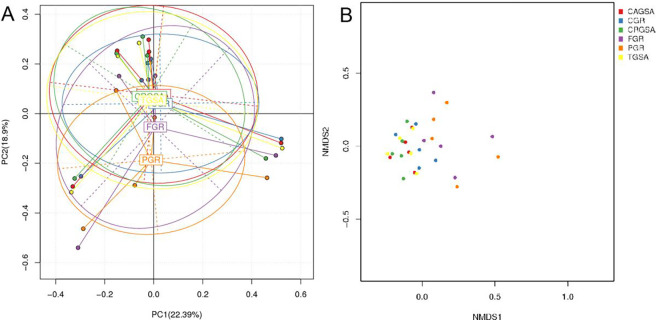
The dots in the figure indicate each sample, and different colors indicate that the samples belong to different groups. 2A: The closer the sample distance, the more similar the composition of the samples. 2B: The closer the distance between the two points of the sample indicates the smaller the difference in community composition between the two.

The analysis of OTU PCA and NMDS showed that the bacterial composition of the cranial glandular sac area (CRGSA), caudal glandular sac area (CAGSA), third glandular sac area (TGSA) and cardiac gland region (CGR) were similar and their bacterial community composition was different. Smaller, and gastric gland area region (FGR) are different, and the pyloric gland area region (PGR) difference is greater. PCA and NMDS analysis results are shown in Fig 2A and 2B.

### 3.2 OTU rank curve analysis

**Fig 3:**
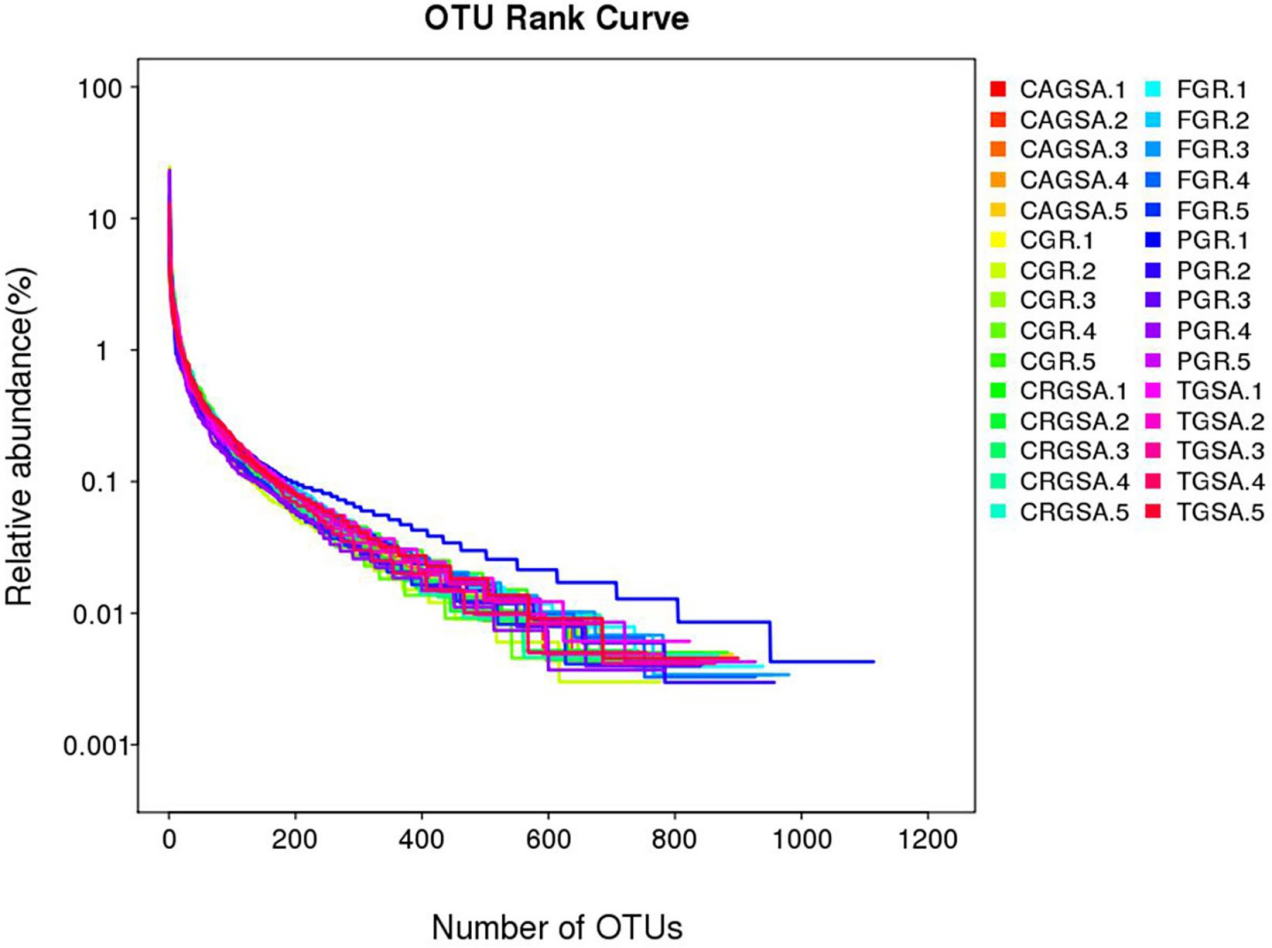
The OUT rank curve. The length of the horizontal axis of the curve reflects the richness of species in the sample, and the wider the curve, the richer the species composition in the sample. The vertical axis of the curve reflects the homogeneity of species in the sample; the wider the curve, the higher the homogeneity of species composition in the sample.

The analysis of the OTU Rank curve shows that the 30 species contained high species richness and high homogeneity of species composition. The subsequent species and abundance analysis can be performed. The results are shown in Fig 3.

### 3.3 Species annotation analysis

**Fig 4:**
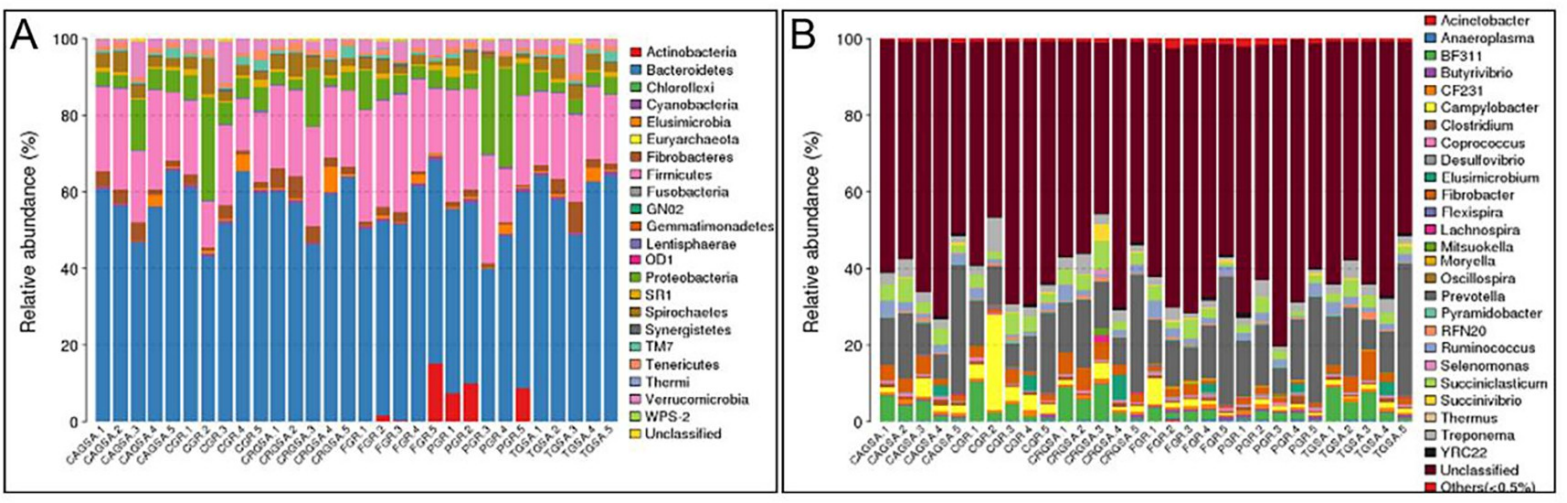
Profiling histogram of water products of sample phylum (A) and genus (B) The different colors represent different species.

Through the comparison with the database Greengene, the species were classified on the OTU, gates, orders, families, genus, and classification levels. A total of 22 samples, 38 classes, 65 orders, 85 families, and 108 genera were identified in 30 samples. At the gate level, *Bacteroidetes*, *Firmicutes* and *Degenerative Bacteria* were predominant, accounting for 54.93%, 21.7%, and 7.3%, respectively. The *Actinomycetes* occupy an important proportion in the fundic gland area and the pyloric gland area. At the genus level: the dominant genus was *Prevotella spp*, *BF311*, *Desmobactobacillus spp*, *Thermospora spp*, *Citrobacter spp*, *Ruminococcus spp*, which accounted for 15.25%, 3.37%, 3.28%, 2.70%, 2.49% and 2.01%, respectively. About 61.36% of bacteria on the genera level have not been identified. The Profiling histogram at the sample gate level and the Profiling histogram for the water sample are shown in Fig 4A and 4B below. (Note: From the outset, all species with species abundance less than 0.5% in all samples will be merged into Other). As shown in Fig 4.

### 3.4 Alpha-Diversity analysis

**Fig 5:**
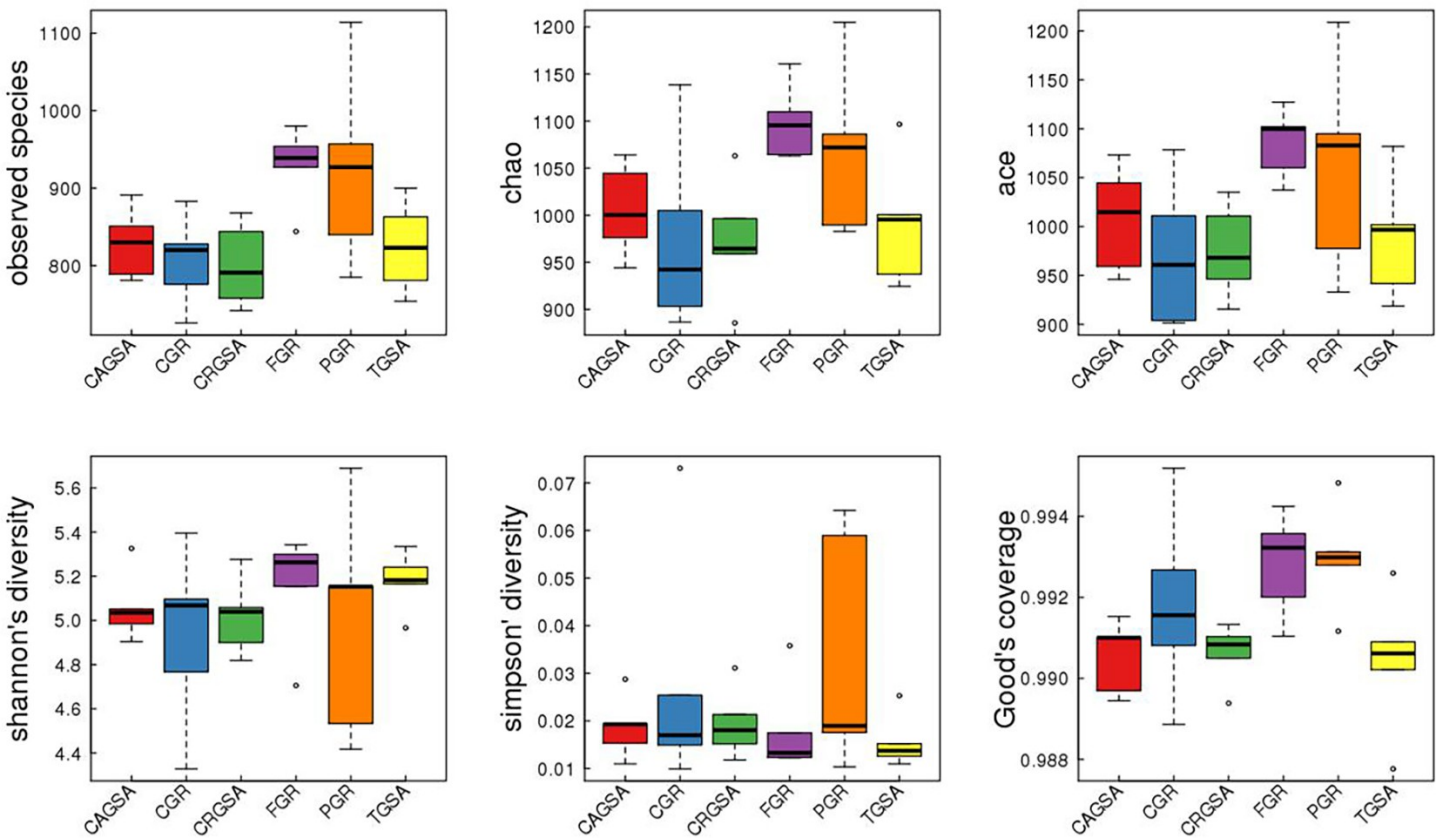
Alpha-Diversity box plot between groups. The box plot shows the minimum value, the first quartile, the median, the third median and the maximum 5 statistics from bottom to top, and "。" indicates an outlier.

Using the inter-group Alpha-Diversity box plot, the abundance of species in the anterior glandular, posterior glandular, third glandular, and cardiac gland areas was lower through the observed species, chao, ace, and shannon index. The richness of species in the glandular and pyloric gland regions is high. (Fig 5)

### 3.5 Analysis and comparison of diversity among samples

**Fig 6:**
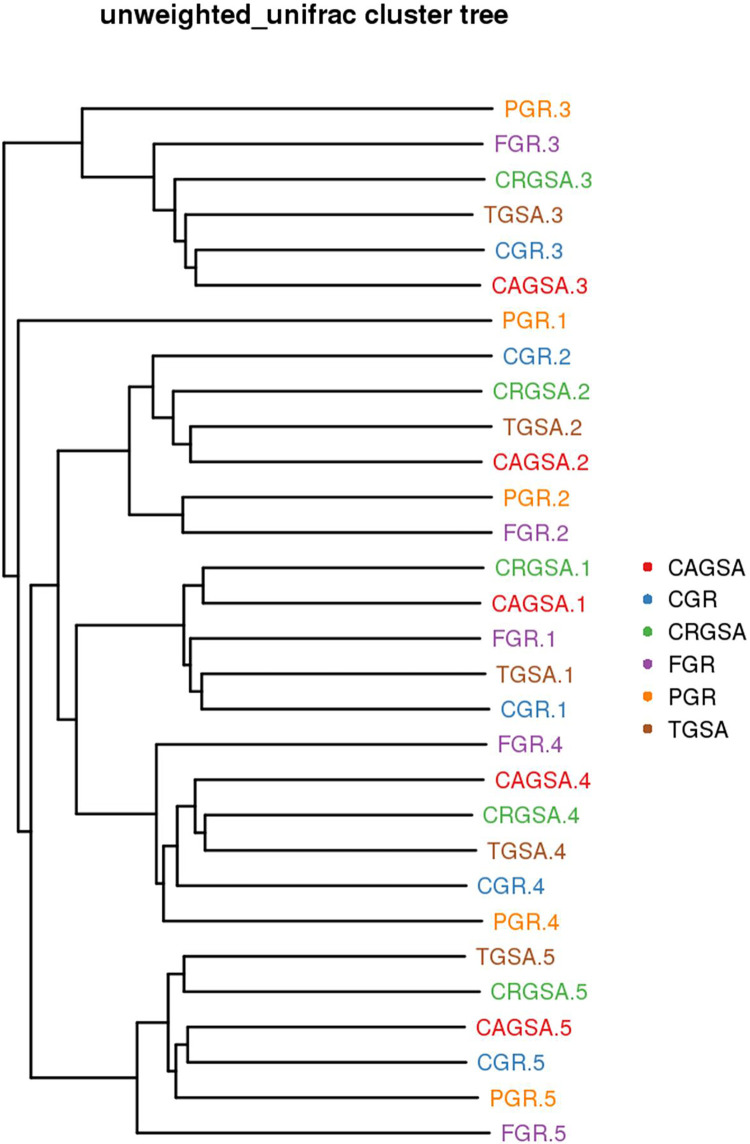
Unweighted-UniFrac index chart. The samples of the same color belong to the same group, and the closer the samples are, the shorter the branch length is, and the more similar the species composition of the two samples is.

The unweighted-UniFrac index was used to measure the differences in species composition between samples. The results showed that the four groups of the anterior sac, the posterior gland sac, the third gland sac, and the cardia adenocarcinoma showed little difference in their species communities, and they were significantly different from the gastric fundic glands and pyloric glands. Gastric glands and pyloric glands have little difference in their species communities. (Fig 6)

### 3.6 Analysis of significant differences between sample groups

**Fig 7:**
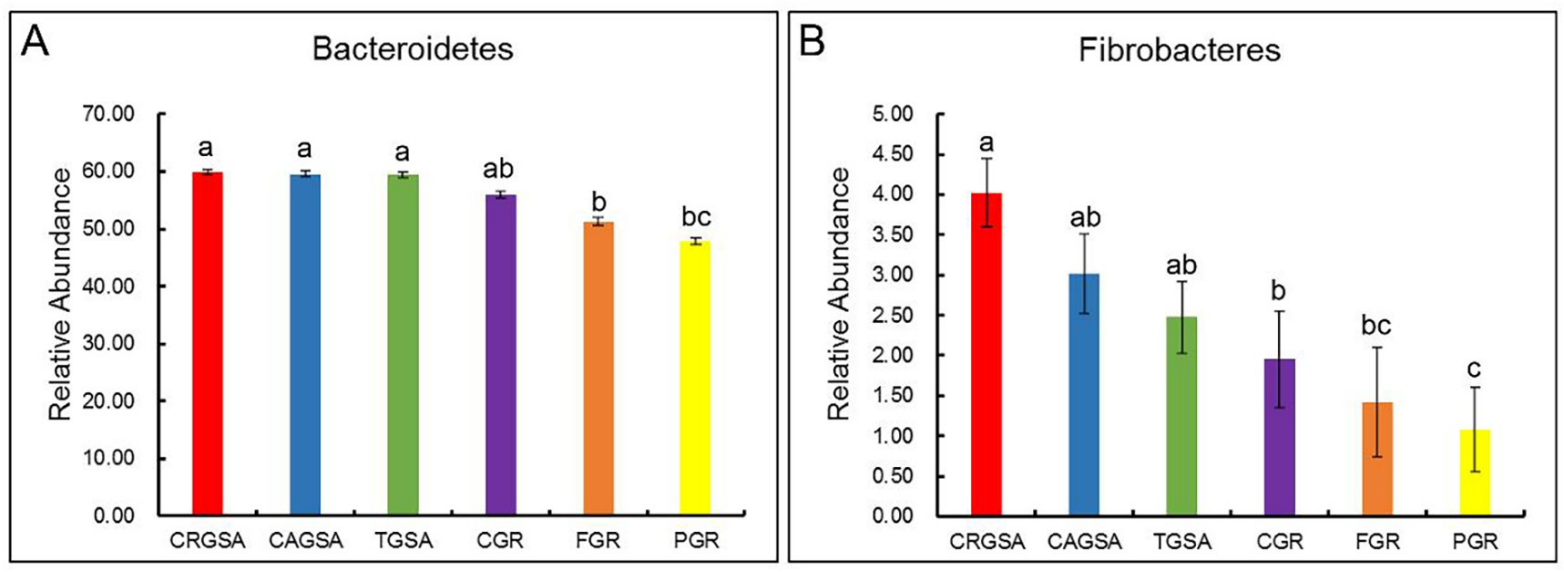
Differences between groups of *Phyllobacterium* and *Fibrobacter*. For each indicator, the same lowercase letters represent no significant difference between groups (*p* > 0.05), while different lowercase letters represent significant difference between groups (*p* < 0.05). The letter "a" represents the maximum value and "a, b, c," in descending order.

The relative abundance of *Bacteroidetes* bacteria in the three glandular cysts was higher than that of the three glands in the abomasum (Fig 7A). The relative abundance of fibroblasts in the glandular sac was higher than that in the abscission gland (Fig 7B). There were 58, 60, 60, 71, 99 and 96 genus CRGSA, CAGSA, TGSA, CGR, FGR and PGR, respectively. The abundance of species in the three glandular sac regions was low and there was no significant difference between them (*p* > 0.05). The glandular area species abundance of the cardia is between the glandular sac area and fundic gland area, and the pyloric gland area. Abundance of abomasum in the abdomen was significantly higher than that in the preglandular sac (*p* < 0.05), and 22 of them were endemic to gastric fundus and cardia. In addition, there are 9 genera in the fundic gland. There are seven genera in the adenocarcinoma of the cardia. Gastric glands and pyloric glands did not differ significantly in the genera.

### 3.7 Histological structural characteristics of different glandular regions of the bactrian camel’s stomach

#### 3.7.1 Submicroscopic structural features of different glandular regions of the stomach

**Fig 8:**
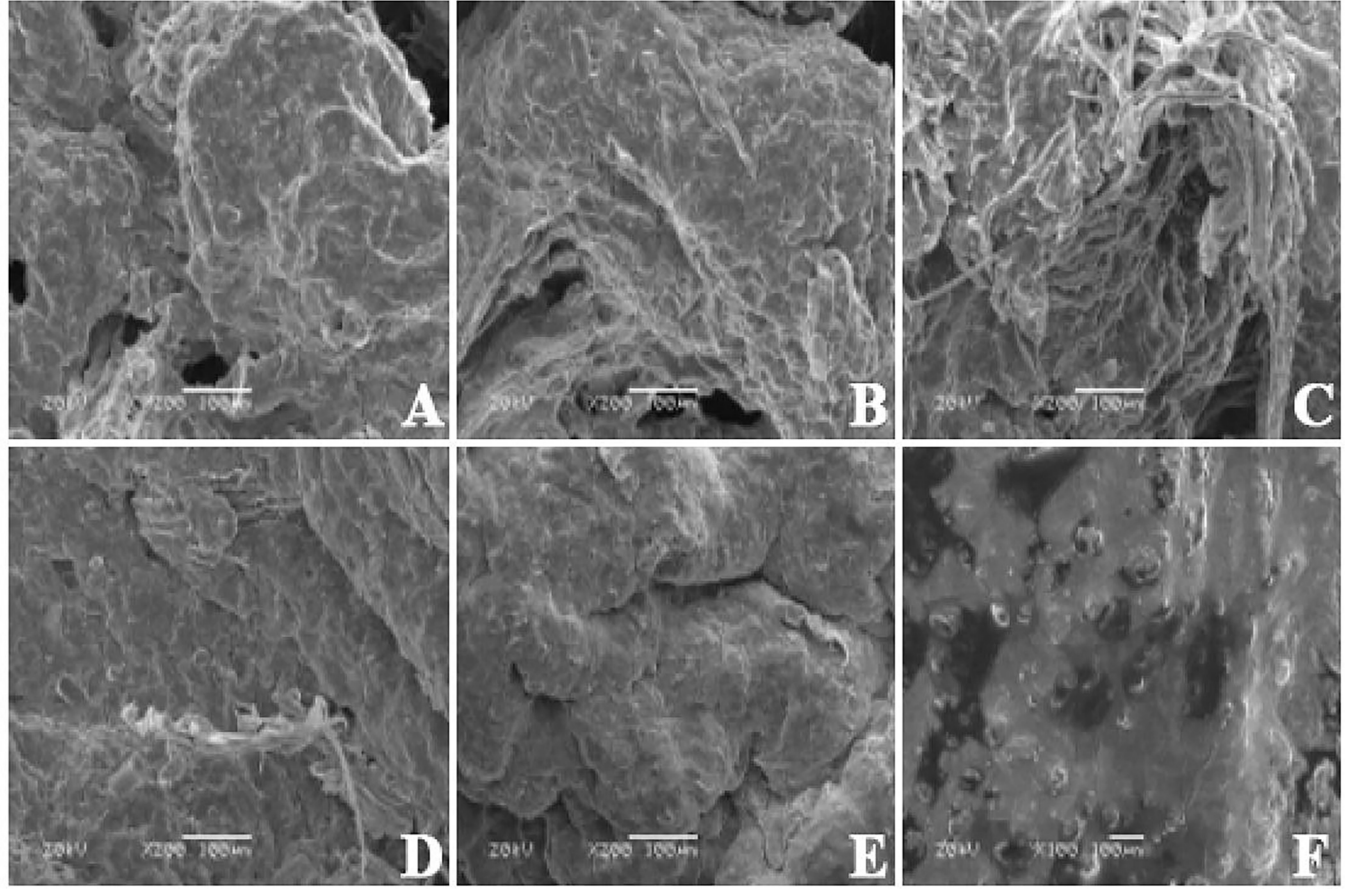
The scanning electron microscope results. A-F are the anterior gland area, posterior gland area, third gland area, cardia gland area, gastric fundic gland area and pyloric gland area.

Scanning electron microscopy revealed that the submicroscopic structures of the anterior glandular capsule, posterior glandular capsule, third glandular capsule, and the mucosal epithelium of the cardia glands of the bactrian camel is similar in textured structure, the mucosa in the region of the fundus and pyloric glands showed longitudinal and transverse ridge-like elevations. (Fig 8)

#### 3.7.2 The AB&PAS staining of different glandular regions of the stomach

**Fig 9:**
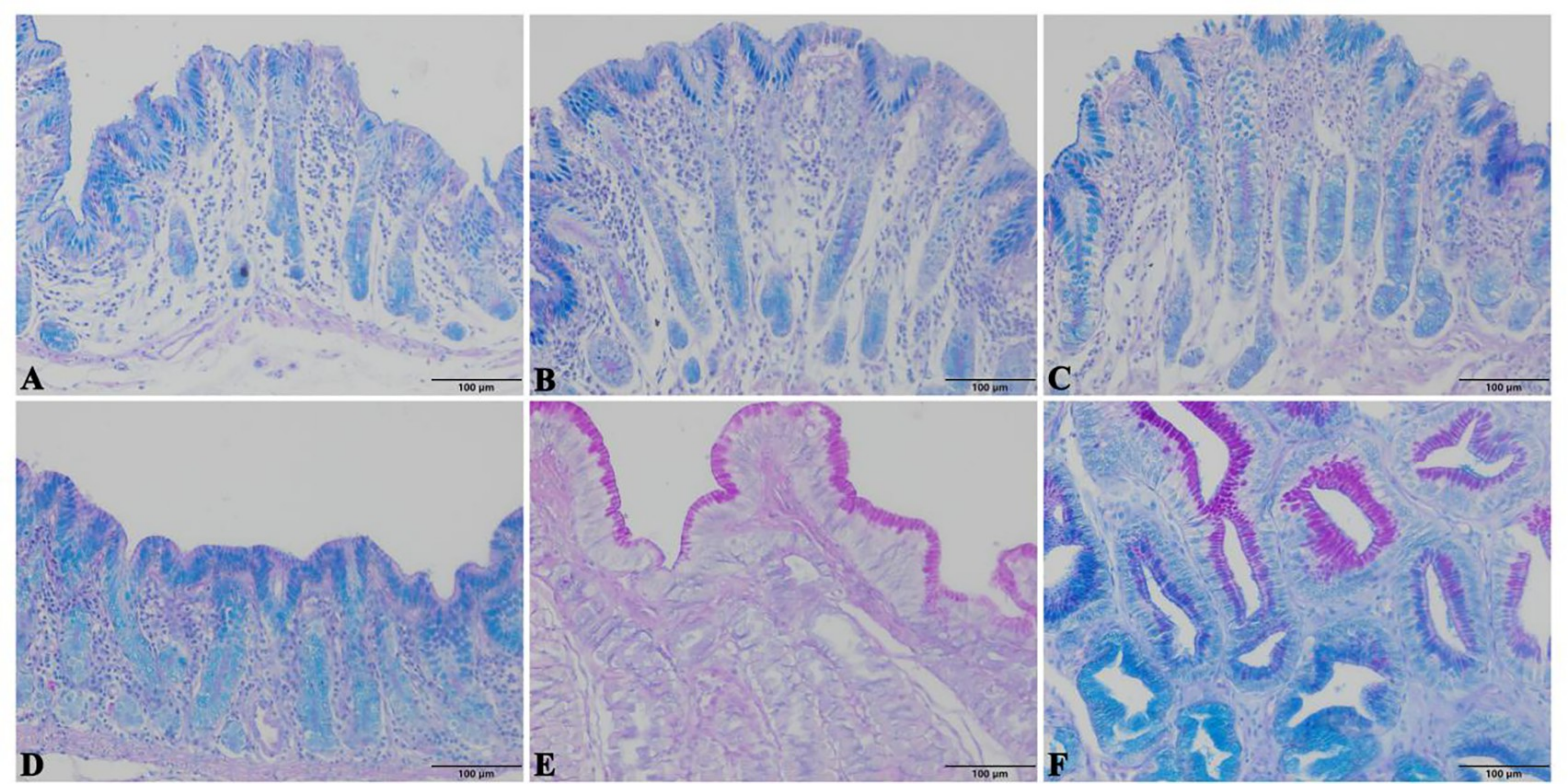
The results of AB&PAS staining in different glandular regions of the stomach. A, B, C, D, E and F indicate the anterior, posterior, third, cardia, fundic, and pyloric gland regions, respectively; neutral, acidic, and moderately acidic mucus is red, blue, and purplish-red, respectively.

AB&PAS staining revealed that: the anterior gland area, posterior gland area, third gland area, and cardia gland area of Bactrian camels mainly secreted acidic mucus, while the gastric fundic gland area mainly secreted neutral mucus and the pyloric region mainly secreted a mixture of acidic and neutral mucus. (Fig 9)

#### 3.7.3 Glandular differences in different glandular regions of the stomach

**Fig 10:**
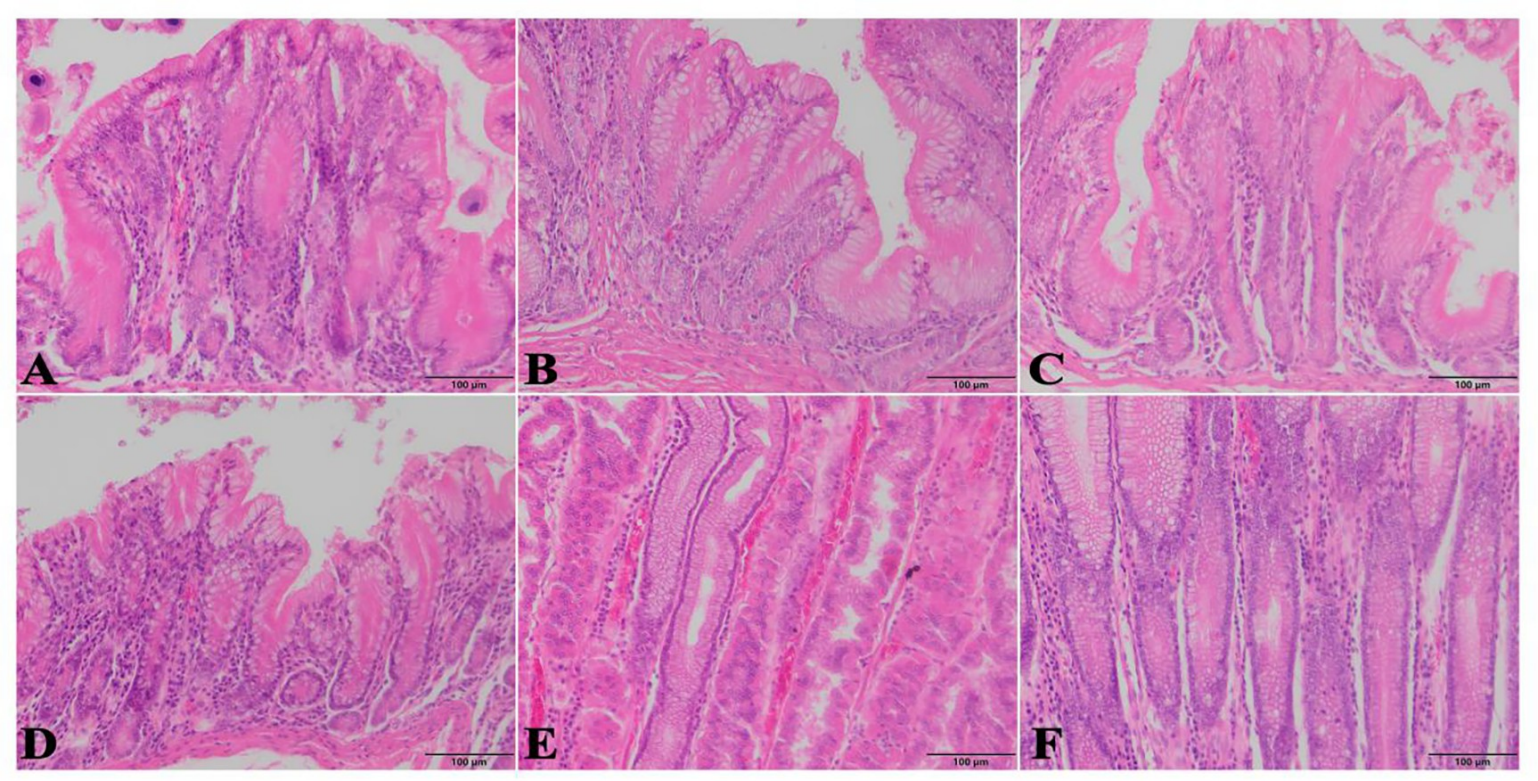
H&E staining of different glandular areas of the stomach. A, B, C, D, E and F indicate the anterior, posterior, third, cardia, fundic, and pyloric gland regions.

The anterior, posterior, third and cardia gland areas are single ductal glands with few branches at the end and some glands are unbranched and reach the mucosal muscle layer. The glands are short, the lumen is small, and the glands open on the mucosal surface through the gastric pit; The epithelium is a columnar epithelium, and as the gland extends to the deep layer of the lamina propria, the height of the epithelium gradually decreases, and the nucleus is located at the base, spherical and densely arranged. The glands of the fundic glands are very long, the gland cavity is small, and some glands are dilated, and there are granular secretions of different sizes in the gland cavity, especially the body and bottom of the gland; The pylorus is rich in pyloric glands, and the superficial layer of the lamina propria is rich in sparse connective tissue, while the deep layer is densely distributed with glands due to the large number of branches of the pyloric glands, and relatively few connective tissues. (Fig 10)

### 3.8. The characterization and differential analysis of IgA+ distribution in different glandular regions of the stomach

#### 3.8.1 The characterization of IgA+ distribution in different glandular regions of the stomach

**Fig 11:**
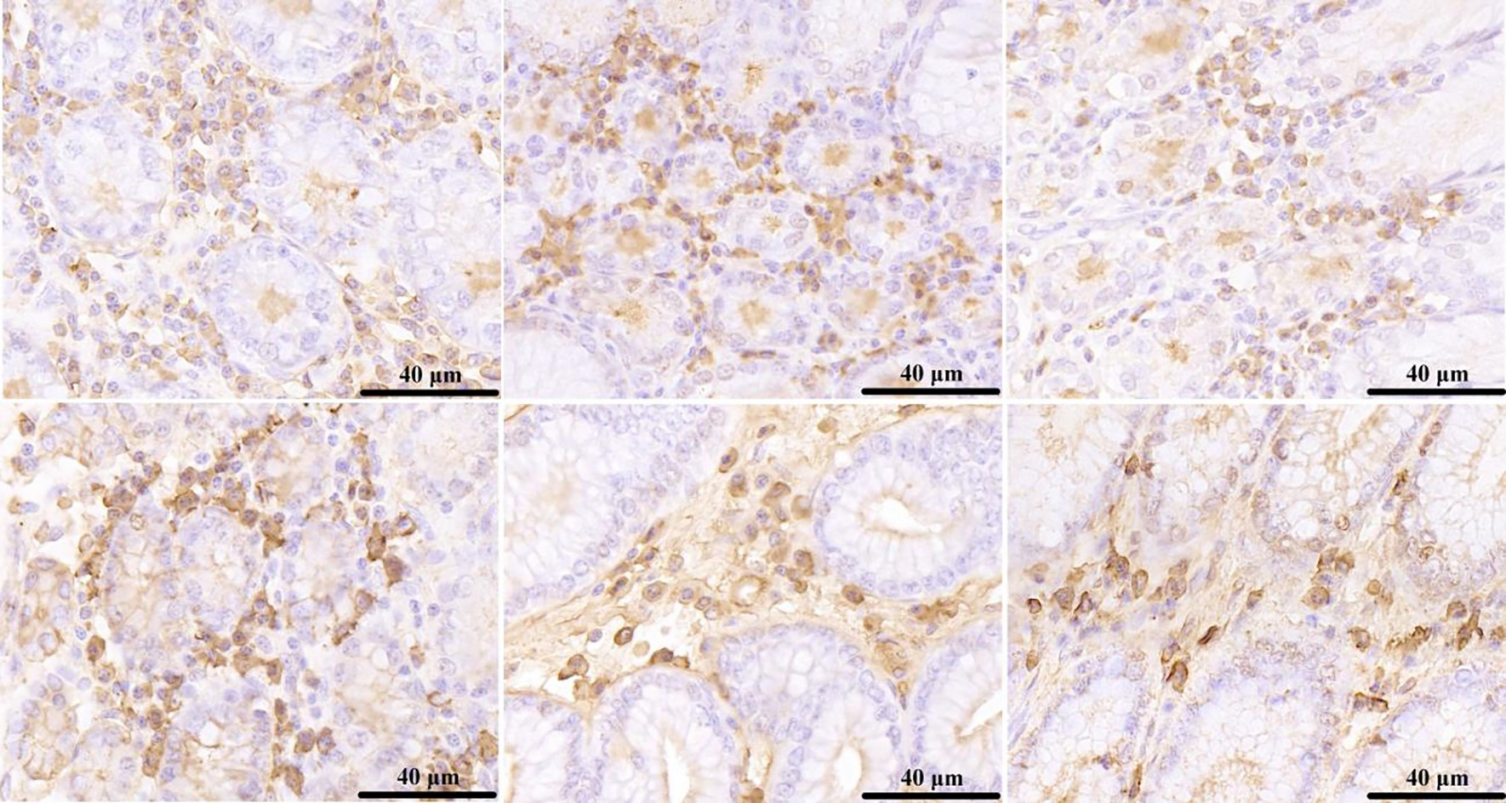
The characterization of IgA+ distribution in different glandular regions of the stomach. A, B, C, D, E and F indicate the anterior, posterior, third, cardia, fundic, and pyloric gland regions.

The results of immunohistochemical staining showed that: IgA+ was secreted by plasma cells, which was a typical secreted protein and expressed in the cytoplasm. IgA+ is mainly distributed around the glands, and the number of anterior glands, posterior glands, third glands, and cardiac glands is more than that of fundic glands and pyloric glands. As shown in Fig 11.

#### 3.8.2 The differential analysis of IgA+ distribution in different glandular regions of the stomach

**Fig 12:**
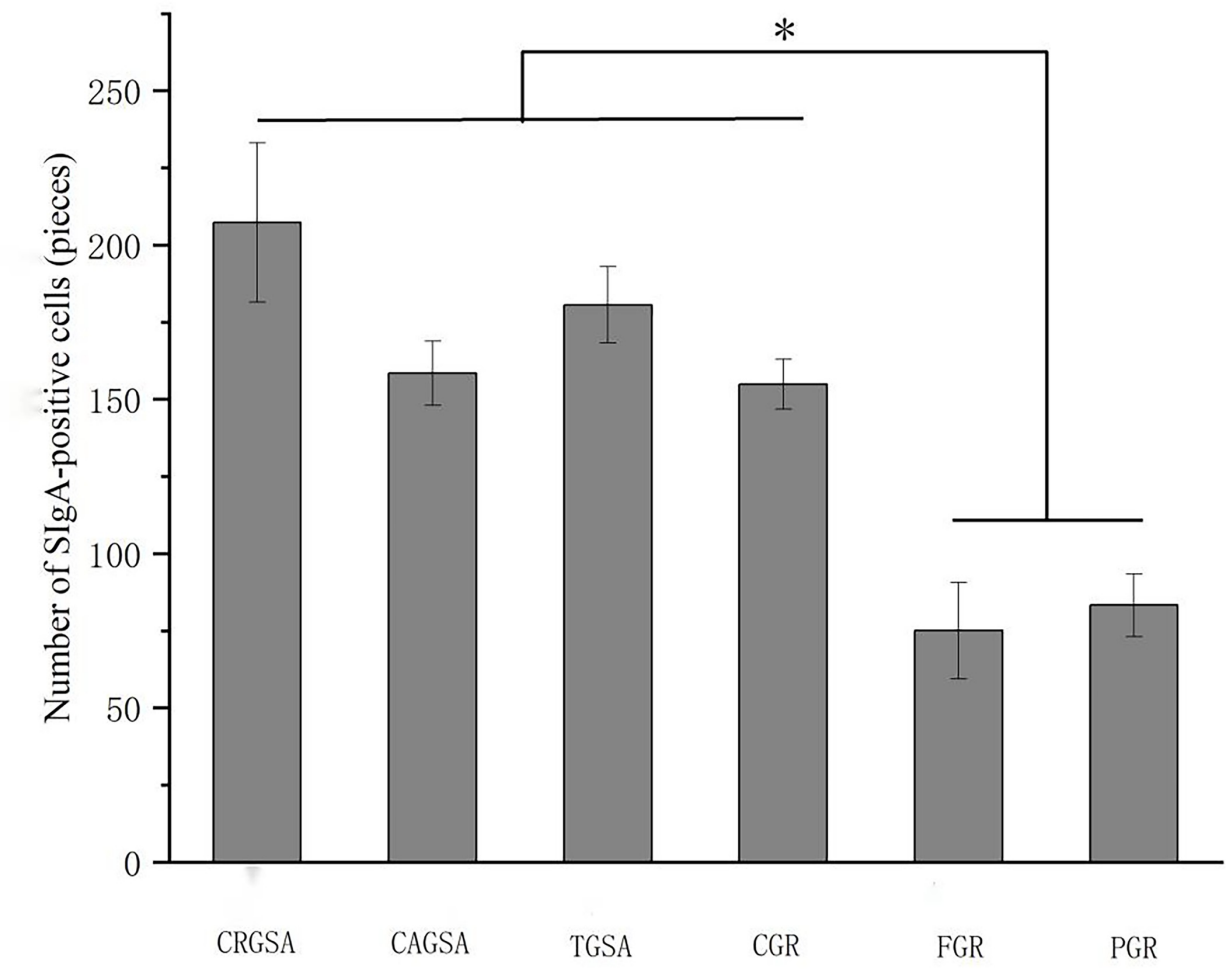
The differential analysis of IgA+ distribution in different glandular regions of the stomach. _*_ indicates significant difference (0.01< *p* <0.05).

Statistical results showed that the number of IgA+ distribution was significantly different in the anterior, posterior, third and cardia glands compared with the fundic and pyloric glands the difference was not significant in the first four glands compared; and the difference was not significant in the last two glands compared. As shown in Fig 12.

## 4. Discussion

The research on influencing the colonization of colonizing microorganisms are currently focused on the following aspects: 1) diet: a recent analysis of the rumen microbiota of 32 animal species showed that the composition of the rumen microbiota is largely determined by diet and may be less influenced by the host, and that the effect of diet on the host is dependent on the microbiota because when dietary components are consumed they are rapidly converted into microbial metabolites [34–41]. 2) Age factor: rumen development with age significantly affects microbial diversity and colonization [42, 43]. 3) Geographic location: a recent global comparative study of the rumen microbiomes of 742 samples from 32 species of animals from 35 countries [44]; although in almost all samples a common bacterial and archaeal core predominated in almost all samples, there were geographic differences in microbial community composition [45]. 4) Genetics and sex also affect microbial colonization to varying degrees [46, 47]. However, there are fewer studies on the influence of the host itself on colonizing microbes.

In this experiment, we found that the proportion of Anaplasma phylum bacteria fixed on the mucosa of three glandular sac areas of Bactrian camel was 59.54%, which was higher than that of rumen contents of Bactrian camel (51%) [48], rumen fluid contents of dromedary camel (55.5%) [49], yak (30.93%) [50] and rumen contents of Jinnan cattle (39.59%) [51]. The percentage of *Fibrobacterium* phylum bacteria in the three glandular sac areas of Bactrian camels was 3.51%, which was higher than that of Qinghai yaks (1.08%) and Gansu yak rumen contents (3.09%). The diet of Bactrian camels consists mainly of hard herbaceous plants with high lignification, ash and cellulose content, so their rumen microorganisms must be capable of degrading these difficult to degrade lignocellulose-rich feeds; *Firmicutes*, *bacteroidetes*, *proteobacteria* and *fibrobacteres* their primary role in the digestive system of ruminants is to break down cellulose and hemicellulose. Species of the genus Proteobacteria have a higher proportion of genes for debranching enzymes and oligosaccharides-degrading enzymes, whereas species of the genera *Fibrobacteria* and *Fibrobacteria* are enriched in cellulases and hemicellulases, and thus these profiles may be key to ensuring lignocellulose degradation. The glandular sac region of the bactrian camel contains a higher proportion of *Anaplasma spp*. and *Fibrobacter spp*. compared to other ruminants, and the high efficiency of cellulose digestion in the Bactrian camel can be attributed to bacteria from these two phyla. Differences in the distribution of bacterial communities in the anterior glandular sac, posterior glandular sac, third glandular sac, cardia, fundus and pyloric gland areas may be related to the tissue structure of the mucosal surface, pH and the amount of IgA+ distribution. Researchers have found that the importance of the mucus layer should not be overlooked, as they have been shown to play a key role in protecting and mediating the microbiota between the CE and the lumen [52]. The pH of different parts of the GI tract also affects the abundance and diversity of the microbial community [53].

The histologic morphology of all four sites, the gastric antral bursa, the posterior bursa, the third bursa and the cardia glands, was similar, and the glands were single-tubular glands with a high number of isolated lymphoid nodules. There were differences in the morphology of the cells of the different glands. The nuclei of the glandular cells of the antrum, posterior bursa, third bursa and cardia glands were located at the basal level, large, rounded, globular, with a distinct nucleolus, and the cytoplasm was basophilic, with the apical part of the deeper glandular cells devoid of eosinophilic granules. The nuclei of the glandular cells of the fundic and pyloric glands were located at the base of the cells, globular or flattened, densely arranged, with very pale H&E staining of the cytoplasm and fine eosinophilic granules. The mucus of the antral, posterior, tertiary and cardia glands was eosinophilic, whereas the mucus secreted by the fundic and pyloric glands was mainly neutral and mixed. It is suggested that glandular cell secretions influence the mucus composition of the mucosa in the gastric glandular region, which in turn affects the pH value of the gastric mucosa and thus participates in the regulation of gastric mucosal function.

In this study, we found that IgA-positive cells were distributed in higher numbers in the anterior glandular capsule, posterior glandular capsule, third glandular capsule, and cardia glands, with a non-significant difference between the four sites when compared to each other (*p* > 0.05), whereas the gastric fundic glands and pyloric glands had a lower number of distribution, with a non-significant difference between the two sites (*p* > 0.05), but the difference between the first four sites and the last two sites was significant when compared to the latter two sites (0.01 < *p* < 0.05). In recent studies it has been shown that IgA and IgG can bind to and influence the colonization of microbial communities [54], and in tissues, IgA is mainly produced as J-chain linked dimers and secreted into the mucus by polymeric Ig receptors. Recent studies have shown that IgA has sufficient affinity to aggregate with microflora through classical agglutination [55, 56]. Typical and atypical binding of IgA to bacterial communities may play a major role in stabilizing bacterial community IgA interactions. It has been found that if the action of IgA decreases or increases the abundance of a key bacterium in the reciprocal metabolic interaction or alters the structure of the bacterial community, it may have a strong indirect effect, which in turn affects the overall microbial community structure [54]. Ruminal epithelial immune development was not altered by different microbial colonization patterns, and the above points are good evidence that IgA has a positive effect on the colonization of colonizing microorganisms. The presence of IgA reduces intestinal pro-inflammatory signaling to mediate intestinal tolerance and modulates the intestinal bacterial composition, while maintaining a dynamic intestinal homeostasis between the host and GIT microorganisms. Host and colonizing microorganisms have a mutualistic relationship, and it has been found that colonizing microorganisms are mainly distributed on the mucus surface [57], and the composition and thickness of mucus affects the microbial population density and diversity [58], and the interaction between host and microorganisms is essentially the development of colonizing microorganisms, which is known as co-evolution [59, 60]. Ruminal pH affects cellulose breakdown by Fibrobacterium cellulolyticum to varying degrees [61]. In this study, we found that different histological and morphological structures of the host rumen, mucus secreted by glands and the structure of colonizing microorganisms in the glandular region were correlated to a certain extent, but their specific interrelationships and mechanisms of action need to be further explored.

Our research has found that the anterior gland sac, posterior gland sac, third gland sac, and cardia gland are single tubular glands with fewer branches at the end, mainly secreting acidic mucus. The gastric fundus gland secretes neutral mucus, while the pyloric gland secretes a mixture of acidic and moderately acidic mucus. There are studies that have shown that the regulation of intestinal microbiota by IgA mainly includes three aspects: changing bacterial motility, regulating gene expression of intestinal bacteria, and assisting in the colonization of some intestinal bacteria [62]. We found that there was no significant difference in the distribution of IgA+in the anterior gland sac, posterior gland sac, third gland sac, and cardia gland, and the distribution of bacterial species was similar. This conclusion confirms that the distribution of IgA+can affect the fixed value of mucosal microbiota. The results of this experiment provide relevant theoretical support for the isolation and cultivation of bacteria that can better degrade cellulose, as well as the reasons for the characterization of microorganisms that affect mucosal characterization.

## 5. Conclusion

The dominant bacterial communities in the glandular area of the stomach of Bactrian camels are *Bacteroidetes* and *Fibrobacteria*, thus possessing stronger ability to digest cellulose. At the same time, it was found that the abundance of colonized microorganisms in the gastric gland area of Bactrian camels may be caused by the pH value of the microenvironment in this area secreted by the glands, as well as the differences in immune regulation of IgA in this area. However, the specific regulatory mechanism still needs further clarification. This study provides a new approach for the study of the impact mechanism of mucosal microbiota colonization, with the aim of providing reference for better utilization and development of mucosal microbiota colonization.
